# Drug-loaded hybrid hydrogels for sonodynamic-chemodyanmic therapy and tumor metastasis suppression

**DOI:** 10.3389/fbioe.2023.1281157

**Published:** 2023-09-18

**Authors:** Xiaoying Wang, Liyun Zhu, Jianhui Zhou, Lingzhou Zhao, Jingchao Li, Changcun Liu

**Affiliations:** ^1^ Office of Hospital Infection and Disease Control and Prevention, Shanghai General Hospital, Shanghai Jiao Tong University School of Medicine, Shanghai, China; ^2^ College of Biological Science and Medical Engineering, Donghua University, Shanghai, China; ^3^ Department of Nuclear Medicine, Shanghai General Hospital, Shanghai Jiao Tong University School of Medicine, Shanghai, China

**Keywords:** alginate hydrogels, drug delivery, tumor metastasis, sonodynamic therapy, chemodyanmic therapy

## Abstract

**Introduction:** Although various therapies have been adopted to treat cancer, metastasis of tumor cells still is a big challenge that compromises therapeutic benefits.

**Methods:** We herein report an injectable drug-loaded hybrid hydrogel that can achieve sonodynamic therapy (SDT) and chemodyanmic therapy (CDT) combined action and suppression of tumor metastasis. This alginate (ALG)-based hydrogel (termed as AMPS) contains manganese dioxide (MnO_2_) nanoparticles as the CDT agents, an organic polymer as the sonosensitizer, and a SIS3 drug as metastasis inhibitor.

**Results:** AMPS is formed via the chelation of ALG by Ca^2+^ in tumor microenvironment, in which MnO_2_ nanoparticles mediate CDT via Fenton-like reaction and the organic polymers enable SDT under ultrasound (US) irradiation by generating singlet oxygen (^1^O_2_), allowing for combinational action of CDT and SDT. In addition, SIS3 is released from AMPS hydrogels to inhibit the metastasis of tumor cells. As such, the AMPS enables a combinational action of SDT and CDT to greatly inhibit the growths of subcutaneous tumors in living mice and also completely suppress the tumor metastasis in lungs and livers.

**Conclusion:** This study thus offers a hybrid hydrogel platform for combinational therapy and metastasis suppression simultaneously.

## 1 Introduction

Sonodynamic therapy (SDT) is a novel therapeutic modality that involves the generation of singlet oxygen (^1^O_2_) for killing malignant tumor cells by irradiation of sonosensitizers by ultrasound (US) (Li et al., 2021; Zhang et al., 2021; Dong et al., 2021; Wang et al., 2022). Compared to traditional therapies such as chemotherapy and radiotherapy, SDT shows unique advantages including high therapeutic selectivity and low side effects (Zhang et al., 2021; Gong and Dai, 2021; Wu et al., 2022; Wang et al., 2023). However, the therapeutic effect of SDT is often limited due to the tumor microenvironment (Tao et al., 2021; Yang et al., 2021; Sun et al., 2022). For example, the hypoxia of tumors often compromises the reactive oxygen species (ROS) generating efficacy of oxygen-dependent SDT (Huang et al., 2020; Geng et al., 2021; Yin et al., 2022). In addition, the high levels of antioxidants, such as glutathione (GSH) in tumor microenvironment will consume the generated ROS, leading to low tumor inhibition rates (Huang et al., 2021; Wu et al., 2022; Kim et al., 2022). Therefore, SDT is often combined with other therapies to improve the antitumor efficacies.

Chemodynamic therapy (CDT) is another type of tumor treatment strategy based on highly toxic hydroxyl radicals (·OH) production by endogenous chemical reactions inside cells to induce cell death (Wang et al., 2020; Tian et al., 2021; Jana and Zhao, 2022; Zhao et al., 2023). In general, Fenton/Fenton-like reactions between CDT agents and hydrogen peroxide (H_2_O_2_) in tumor microenvironment (TME) are considered as the main characters of CDT (Zhang et al., 2021; Hao et al., 2021; Jia et al., 2022). Because Fenton/Fenton-like reactions rely on the levels of H_2_O_2_ in the disease sites, and thus CDT also has the advantages of high treatment selectivity and low side effects (Wang et al., 2020; Li et al., 2021; Huang et al., 2022). Currently, various nanoplatforms have been developed and used as CDT agents for tumor CDT (Meng et al., 2020; Zhou et al., 2021; Cao et al., 2022). In addition, CDT can amplify oxidative stress and consume GSH by producing ROS, and thus the combination of CDT and SDT has been a promising strategy for cancer therapy (Zhao et al., 2022; Chen et al., 2022; Liu et al., 2023).

Tumor metastasis is a certain course involving the spreads of some malignant tumor cells, such as lung cancer, breast cancer, melanoma and colon cancer (Zhang et al., 2020). Tumor metastasis is the leading cause of cancer treatment failure and death of patient, while its diagnosis and therapy are a great challenge (Fan et al., 2019; Ling et al., 2020; Gao et al., 2021). Various therapeutic strategy such as chemotherapy, SDT and CDT can only ablate local tumors, but they fail to deal with tumor metastasis if the local tumors are not completely eradicated (Ding et al., 2022). Therefore, suppression of tumor metastasis is pivotal after cancer treatment to achieve desired therapeutic benefits. The interventions of tumor metastasis using drug-loaded nanoparticles have been reported. For example, a SIS3-loaded nanoscale polymer prodrug was designed to achieve photodynamic therapy-enhanced chemotherapy and tumor metastasis inhibition (Cheng et al., 2022). However, the drug delivery efficacies into tumor sites using nanoparticles are often poor and the uncontrolled releases of drugs will lead to side effects (Cheng et al., 2022). Alternatively, hydrogels are increasingly studied for drug delivery because of their high drug loading efficacy and local drug release property (Wang et al., 2022; Zhao et al., 2022; Sood et al., 2023). The uses of hydrogels to deliver drugs for combined cancer therapy and tumor metastasis inhibition have been poorly explored.

**FIGURE 1:**
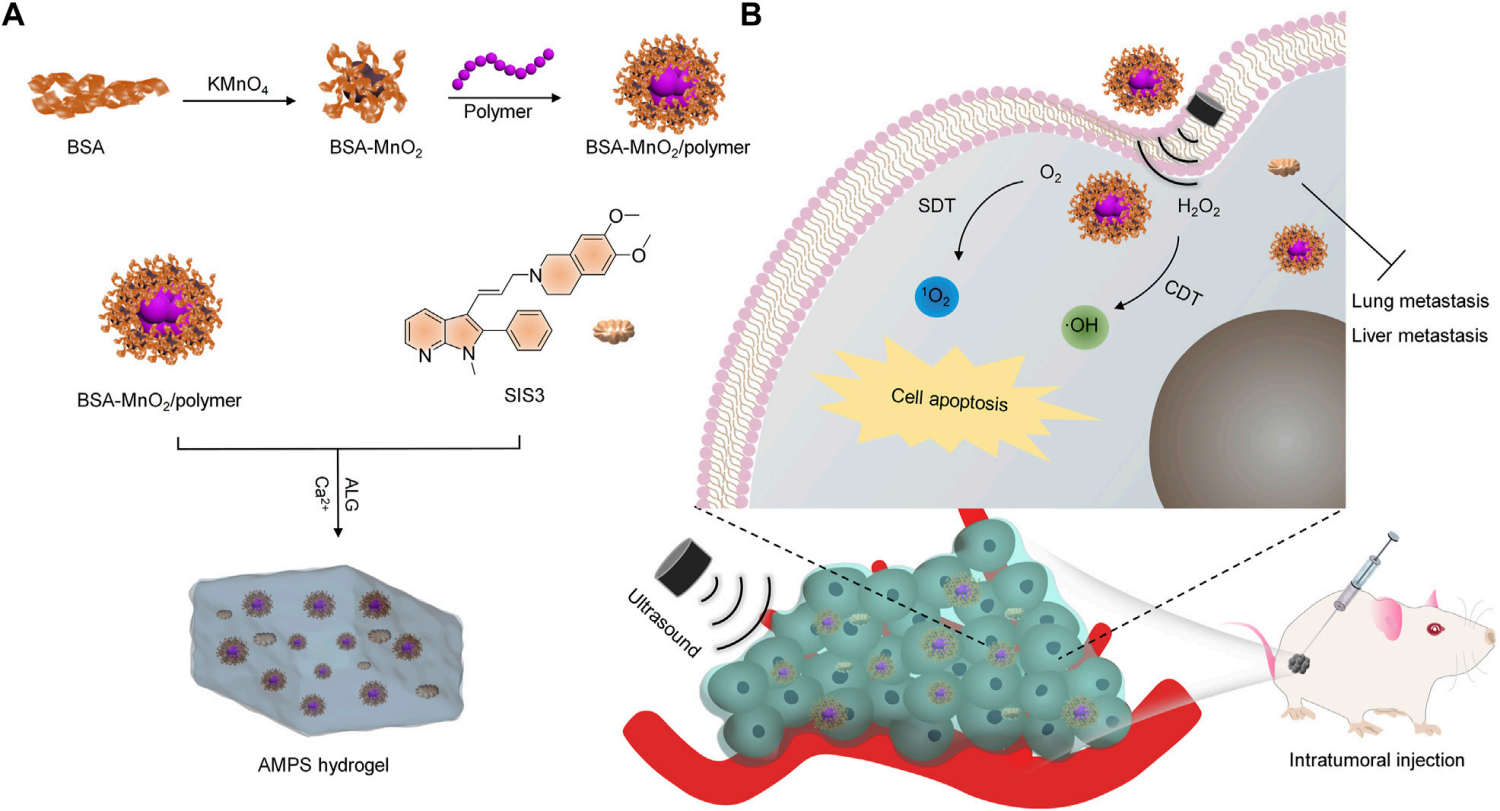
Schematic illustration of AMPS hydrogels for SDT-CDT combinational therapy and tumor metastasis inhibition. **(A)** The fabrication of the AMPS hydrogels. **(B)** Schematic illustration of cancer treatment using AMPS hydrogels via SDT-CDT combinational therapy and tumor metastasis inhibition.

In this study, an alginate (ALG)-based hydrogel platform is developed for SDT-CDT combined therapy of breast cancer and inhibition of tumor metastasis. ALG hydrogels can be formed through the cross-linking of ALG by Ca^2+^, and show the advantages of simple synthesis, injectability, and good biocompatibility (Ding et al., 2022). The ALG hydrogels (termed as AMPS) were loaded with manganese dioxide (MnO_2_) nanoparticles as the CDT agents, an organic polymer as the sonosensitizer for SDT, and SIS3 as a therapeutic drug for inhibiting tumor metastasis, which were *in-situ* formed via coordination of ALG by Ca^2+^ (Figure 1A). In the tumor sites with irradiation of ultrasound (US), AMPS produced ^1^O_2_ and OH via SDT and CDT effect, respectively to enable the killing of tumor cells (Figure 1B). Moreover, the released SIS3 from AMPS could disturb the migration of tumor cells and thus resulted in tumor metastasis inhibition in lungs and livers. As such, the AMPS treatment with US irradiation of tumors achieved obvious inhibition of tumor growths and suppression of tumor metastasis in subcutaneous 4T1 tumor-bearing mouse models.

## 2 Materials and methods

### 2.1 Materials

Bovine serum albumin (BSA), sodium alginate and anhydrous CaCl_2_ were obtained from Aladdin Reagent Co. Ltd. (Shanghai, China). Organic polymer, 2′,7′-dichlorofluorescin diacetate (H_2_DCFH-DA), and singlet oxygen sensor green (SOSG) were obtained from Sigma-Aldrich (St. Louis, United States). SIS3 was purchased from Selleck (Shanghai, China).

### 2.2 Material characterization

A Zetasizer Nano-series (Nano-ZS90, Malvern, UK) was used to measure nanoparticle sizes and zeta potentials. A scanning electron microscope (SEM, SU8010, Japan) was used to obtain the images. A fluorescence spectrophotometer (RF-6000, SHIMADZU, Japan) and absorbance spectrophotometer (Persee, TU-1810, Beijing, China) was used to obtain fluorescence and UV-vis spectra, respectively.

### 2.3 Synthesis of BSA-MnO_2_/polymer nanoparticles

A solution of KMnO_4_ (31.6 mg) was mixed with the BSA solution (250.0 mg), and the obtained solution was stirred at 37°C for 2 h. After purification via dialysis using dialysis bag (the molecular weight cut-off = 8–14 kDa), BSA-MnO_2_ were obtained. Then 1 mL of tetrahydrofuran containing the organic polymer (0.25 mg) was dropped into the BSA-MnO_2_ solution, and the solution was sonicated for 5 min. After purification via ultrafiltration, BSA-MnO_2_/polymer nanoparticles were obtained.

### 2.4 Synthesis of hydrogels

Sodium alginate solutions containing BSA-MnO_2_/polymer nanoparticles and SIS3 were slowly injected into a solution of CaCl_2_ at the final Ca^2+^ concentration of 1.8 mM (the Ca^2+^ concentration in extracellular microenvironment of living tissues), and then AMPS hydrogels were formed. Similarly, sodium alginate solutions containing BSA-MnO_2_/polymer nanoparticles were slowly injected into the CaCl_2_ solution to synthesize the hydrogels without SIS3 loading (termed as AMP) and used as control.

### 2.5 Sonodynamic effect evaluation

The frequency and power density of US in all experiments was set as 1.0 MHz and 1.0 W/cm^2^, respectively. The solutions of AMPS and AMP were mixed with a ^1^O_2_ probe (SOSG), and the mixed soultions were treated by US irradiation for different time. Fluorescence spectra of SOSG were measured using fluorescence spectrophotometer to evaluate the ^1^O_2_ generation.

### 2.6 Cell viability analysis

4T1 cancer cells were treated with AMPS and AMP hydrogels at different concentrations for 24 h, and then the cells were carefully washed to remove the hydrogels. The cell viability was evaluated using cell counting kit-8 (CCK-8) analysis.

### 2.7 Evaluation of ROS levels

4T1 cancer cells were treated with AMPS and AMP hydrogels in the presence of H_2_O_2_ (100 μM), and then the cells were treated with H_2_DCFDA (10 μM) for 30 min. After US irradiation of the cells, fluorescence images of the treated cells were obtained.

### 2.8 Evaluation of *in vitro* therapeutic efficacy

4T1 cancer cells were treated with AMPS and AMP hydrogels in the presence of H_2_O_2_ (100 μM) for 24 h. After US irradiation, the cell viability was analyzed using CCK-8 analysis.

### 2.9 Dead/living cell staining

4T1 cells were treated with AMPS and AMP hydrogels, followed by US irradiation of 4T1 cells. Then the cells in each group were stained with calcein-AM and propidium iodide (PI) to obtain the fluorescence images of cells.

### 2.10 Wound healing analysis

After culture of 4T1 cells, the scratches of 4T1 cells were made in cell culture flasks. The cells were then incubated with AMPS and AMP hydrogels, followed by US irradiation. At different times, the images of cell scratches were captured using microscopy.

### 2.11 4T1 tumor mouse model establishment

All animal experiments in this study were conducted with the permissions by Animal Care and Use Committee of Donghua University. Subcutaneous 4T1 tumor-bearing mouse models were established using female nude mice (3–4 weeks, around 15.0 g).

### 2.12 *In vivo* therapeutic efficacy evaluation

4T1 tumor-bearing mouse models were treated with PBS, AMPS and AMP hydrogels, and then the tumors were irradiated by US for 10 min. After different treatments, the sizes of tumors and body weights of mice were measured for 24 days. The tumor volumes and relative tumor volumes were calculated to evaluate the antitumor efficacy. On day 24, the tumors were collected from mice in each group and the tumor weights were measured.

### 2.13 Histology staining

On day 24, the mice after treatments were euthanized and tumors were collected from mice. The tumors were used for histological analysis via hematoxylin and eosin (H&E) staining. The staining images were captured using microscope.

### 2.14 *In vivo* metastasis inhibition evaluation

4T1 tumor-bearing mouse models were treated with PBS, AMPS and AMP hydrogels, and the tumors were irradiated by US for 10 min. After 30 days of treatments, lungs and livers were collected form mice. The metastatic tumor nodules in lungs and livers were evaluated using H&E staining.

### 2.15 Statistical analysis

All trials were duplicated for at least three times and the figures in this paper were expressed as average ± standard deviation. The significant difference was indicated as * *p* < 0.05, ** *p* < 0.01, and *** *p* < 0.001, respectively.

## 3 Results and discussion

### 3.1 Preparation and characterization of hydrogels

**FIGURE 2:**
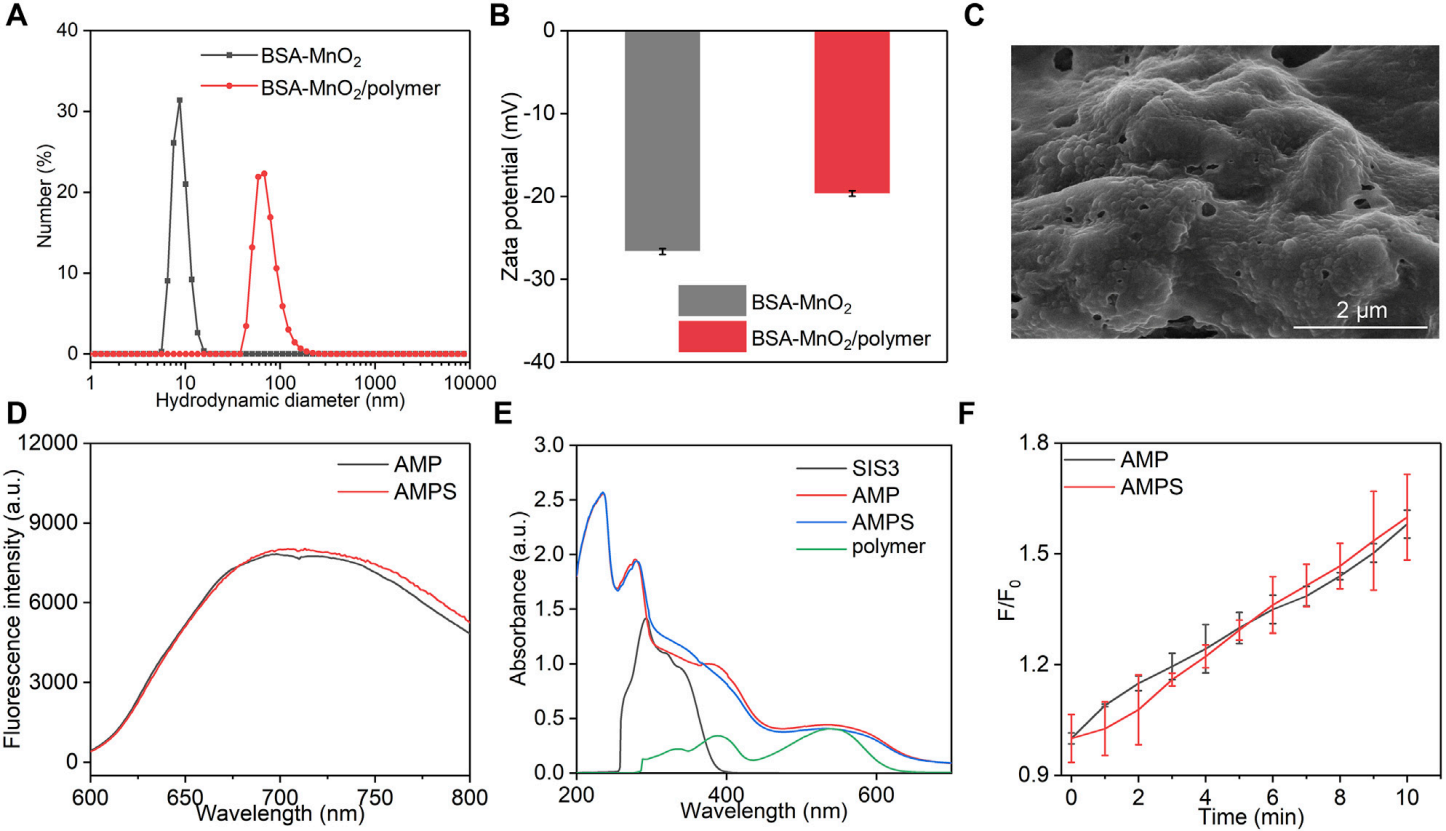
Characterization of AMPS and AMP hydrogels. **(A)** Hydrodynamic sizes of BSA-MnO_2_ and BSA-MnO_2_/polymer nanoparticles. **(B)** Zeta potentials of BSA-MnO_2_ and BSA-MnO_2_/polymer nanoparticles. **(C)** SEM image of AMPS hydrogels. **(D)** Fluorescence spectra of AMPS and AMP hydrogels. **(E)** Absorbance spectra of SIS3, polymer, AMPS and AMP hydrogels. **(F)** ^1^O_2_ generation for AMPS and AMP hydrogels under US irradiation for different times.

To synthesize AMPS and AMP hydrogels, BSA-MnO_2_ and BSA-MnO_2_/polymer nanoparticles were synthesized. The hydrodynamic size of BSA-MnO_2_/polymer nanoparticles was larger than that of BSA-MnO_2_ (Figure 2A). BSA-MnO_2_ and BSA-MnO_2_/polymer nanoparticles similarly showed a negative surface potential (Figure 2B). AMPS hydrogels were formed via injecting ALG solution containing BSA-MnO_2_/polymer nanoparticles and SIS3 into Ca^2+^ solution. The injection of ALG solution containing only BSA-MnO_2_/polymer nanoparticles into Ca^2+^ solution led to the formation of control hydrogels (AMP).

As shown in SEM image, a cross-linked surface network structure was observed for AMPS hydrogels with surface loading of nanoparticles (Figure 2C). AMPS and AMP hydrogels similarly showed a fluorescence emission at around 700 nm (Figure 2D). A characteristic peak of polymer at around 560 nm could be observed in the absorbance spectra of AMPS and AMP hydrogels, which however was not detected for SIS3 (Figure 2E). These results suggested that AMPS and AMP hydrogels had similar optical properties. The sonodynamic effect of AMPS and AMP hydrogels was then investigated by measuring the generation of ^1^O_2_. Under US irradiation, the fluorescence intensity of solutions containing ^1^O_2_ probe and hydrogels gradually increased, suggesting the generation of ^1^O_2_ (Figure 2F). After 10 min of US irradiation, the fluorescence intensity enhancement was similar for AMPS and AMP hydrogels. Thus, these two hydrogels had consistent ^1^O_2_ generation efficiency.

### 3.2 *In vitro* ROS generation and therapeutic efficacy

**FIGURE 3:**
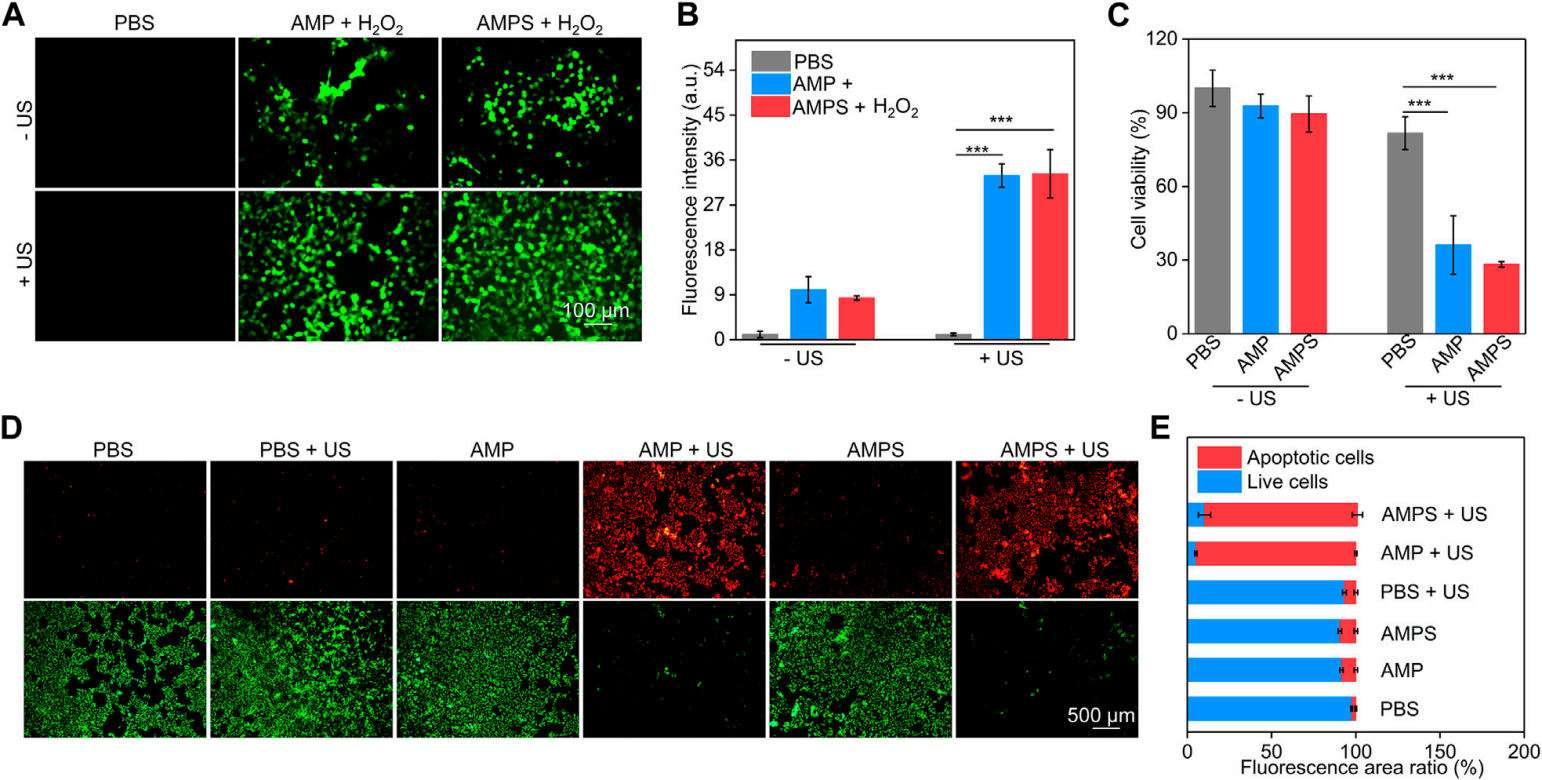
*In vitro* ROS generation and therapeutic efficacy. **(A)** Fluorescence images of ROS inside 4T1 cells after incubation with AMP and AMPS hydrogels plus US irradiation. **(B)** Fluorescence intensity of green signals of the generated ROS, *** *p* < 0.001. **(C)** Cell viability of 4T1 cells after AMP and AMPS hydrogel treatment plus US irradiation, *** *p* < 0.001. **(D)** Living/dead staining images of 4T1 cells in various groups. **(E)** Fluorescence area ratios of red fluorescence signals (apoptotic cells) and green fluorescence signals (live cells).

To verify the cancer cell killing ability of AMPS and AMP hydrogels, the ROS generation inside 4T1 cells was evaluated. In AMP + H_2_O_2_ and AMPS + H_2_O_2_ groups, obvious green fluorescence signals could be detected due to the generation of OH via CDT effect, however, the green fluorescence signals were not observed in PBS group (Figure 3A). Moreover, much stronger green fluorescence signals were observed in AMP + H_2_O_2_ + US and AMPS + H_2_O_2_ + US groups, which was due to the generation of OH and ^1^O_2_ via CDT and SDT effects. The fluorescence intensities of ROS signals in cells of AMP + H_2_O_2_ + US and AMPS + H_2_O_2_ + US groups were higher than those in cells of AMP + H_2_O_2_, AMPS + H_2_O_2_ and PBS groups (Figure 3B). These results verified the ROS generation inside cells after hydrogel treatment and US irradiation. The generated ROS via CDT and SDT effect would consume glutathione (GSH) in cancer cells, thus improving the therapeutic effect compared to sole CDT or SDT.

CCK-8 assay was used to evaluate the cell cytotoxicity and *in vitro* therapeutic efficacy. 4T1 cells were treated with AMPS and AMP hydrogels at different concentrations for 24 h, and their cell viability did not have obvious changes compared to that of control cells (Supplementary Figure S1, supporting information). This confirmed the low cytotoxicity of AMPS and AMP hydrogels. In AMPS + US and AMP + US groups with the addition of H_2_O_2_, the cell viability of 4T1 cells was obviously reduced, confirming the therapeutic efficacy (Figure 3C). The cell viability in these two groups was similar due to their consistent *in vitro* therapeutic efficacy. Living and dead staining images showed that some dead cells with red fluorescence signals could be found in AMPS- and AMP-treated groups compared to PBS group (Figure 3D). Much stronger red fluorescence signals were observed in AMP + US and AMPS + US groups and only a small numbers of living cells (green fluorescence signals) were observed in these two groups. The fluorescence area ratio of apoptotic cells in AMP + US and AMPS + US groups was much higher than that in the other groups (Figure 3E). These results verified the good *in vitro* therapeutic efficacy of AMP and AMPS hydrogels with US irradiation.

### 3.3 *In vivo* antitumor efficacy evaluation

**FIGURE 4:**
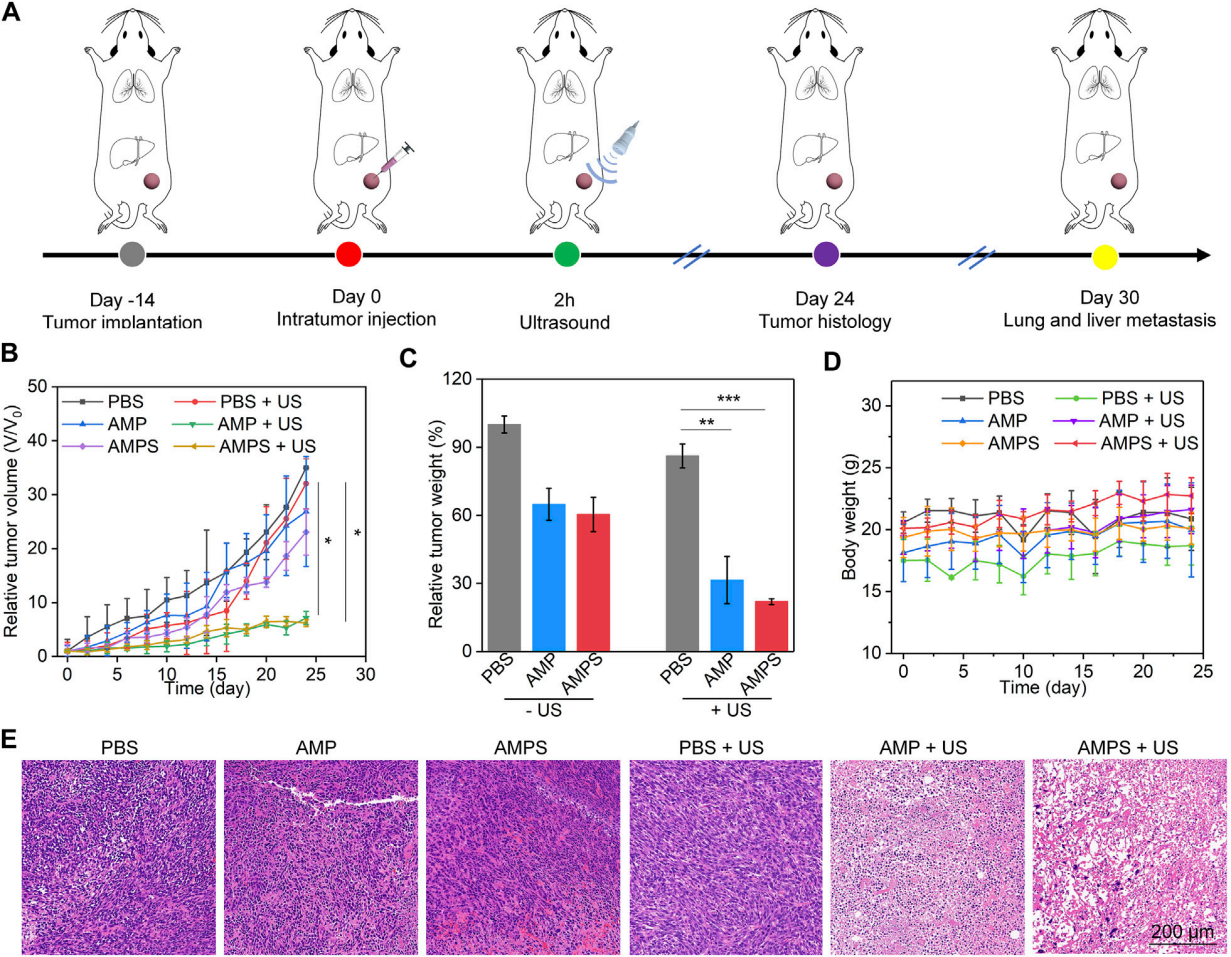
*In vivo* antitumor efficacy evaluation. **(A)** Schematic of antitumor efficacy evaluation via treating tumors with hydrogels and US irradiation. **(B)** Relative volumes of 4T1 tumors in each group, * *p* < 0.05. **(C)** Relative tumor weights of 4T1 tumors in each group, ** *p* < 0.01, *** *p* < 0.001. **(D)** Body weights of 4T1 tumor-bearing mice after various treatments. **(E)** H&E staining images of tumors from various treated mice.

4T1 tumor-bearing mouse models were used to evaluate the *in vivo* therapeutic efficacies. The tumors were treated with PBS, AMP and AMPS hydrogels, followed by US irradiation (Figure 4A). In the treated mice, the tumor growths were lower than those in PBS control mice (Figure 4B). The slight inhibition of tumor growths in AMP- and AMPS-treated groups was due to the CDT effect, while the obvious tumor inhibition efficacies in AMP + US and AMPS + US groups was because of the CDT and SDT combination effect. The relative tumor volumes in AMP + US and AMPS + US groups were similar after 24 days of treatments. The tumor weights in AMP, AMPS, AMP + US and AMPS + US groups were lower than those in PBS and PBS + US groups (Figure 4C). In addition, the tumors in AMP + US and AMPS + US groups had lower weights compared to those in AMP and AMPS groups. The results verified the good *in vivo* antitumor efficacy of AMP and AMPS with US irradiation. The body weights of tumor-bearing mice after treatments were stable and did not have obvious changes in each group (Figure 4D). As shown in H&E staining images, heart, spleen and kidney of mice in AMPS + US group showed similar histological morphologies as those in PBS group (Supplementary Figure S2, supporting information). Therefore, the AMPS hydrogel-mediated treatment had good *in vivo* biosafety.

H&E staining of tumors was then carried out to further confirm the antitumor efficacy. As shown in the staining images, cell apoptosis and necrosis could be observed in AMP, AMPS, AMP + US and AMPS + US groups, but not in PBS and PBS + US groups (Figure 4E). The levels of cell apoptosis and necrosis in AMP + US and AMPS + US groups were much higher than those in AMP and AMPS groups. These results indicated the similar therapeutic efficacy in AMP + US and AMPS + US groups.

### 3.4 *In vivo* anti-metastasis investigation

**FIGURE 5:**
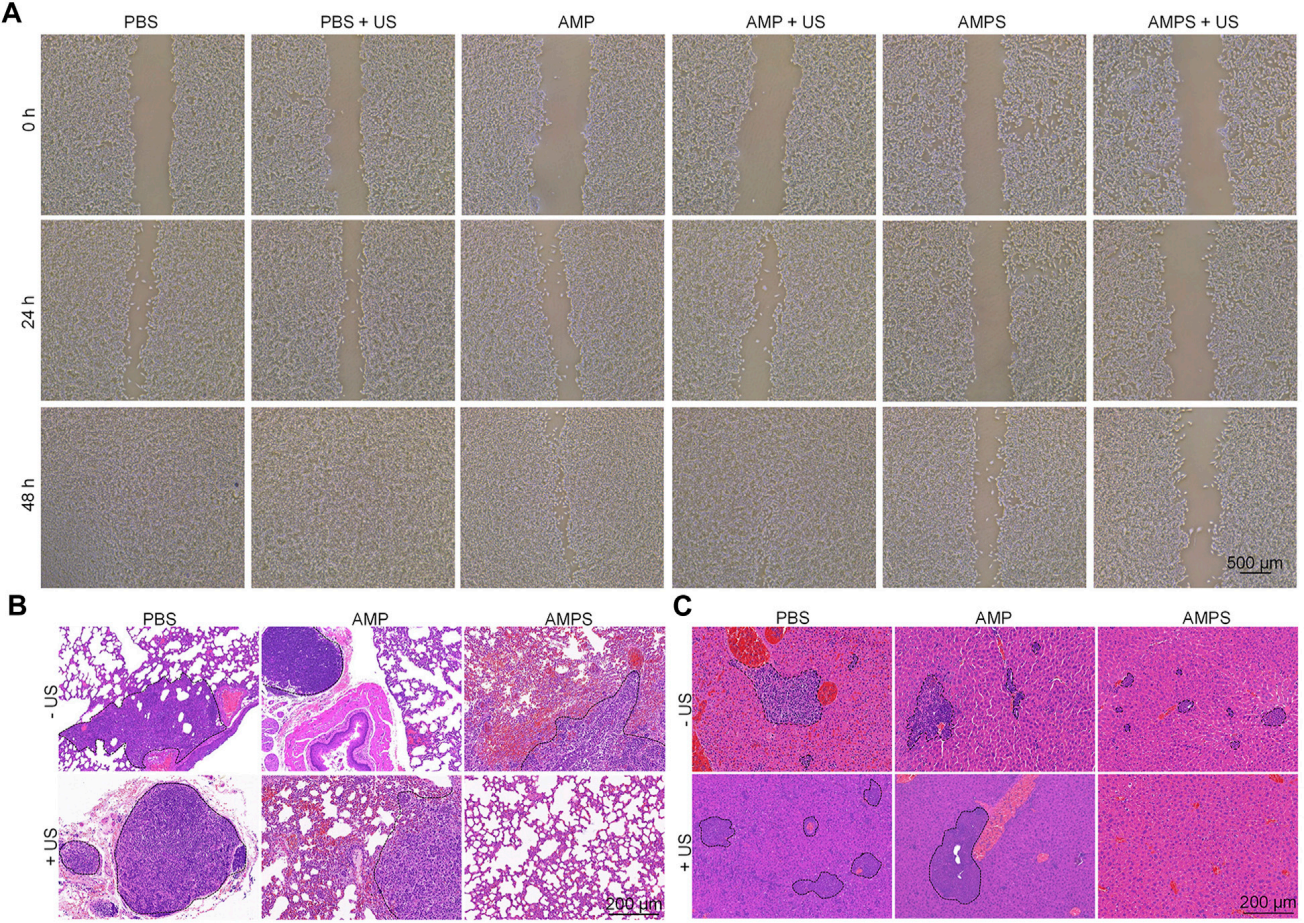
*In vivo* anti-metastasis investigation. **(A)** Pictures of scratches of 4T1 cells in various treated groups. **(B)** H&E staining images of lungs from various treated mice. **(C)** H&E staining images of livers from various treated mice.

To verify the inhibition of cancer cell migration by AMPS hydrogels, *in vitro* wound healing experiments were carried out (Shi et al., 2022; Sun et al., 2023). As shown in the pictures, breadths of cell scratches gradually reduced and were almost vanished after 48 h of culture in AMP, AMP + US, PBS and PBS + US groups (Figure 5A), which was due to the migration of 4T1 cancer cells after culture. However, the cell scratches were still observed in AMPS and AMPS + US groups even after 48 h of culture. These results suggested that the cell migration could be obviously inhibited by AMPS hydrogel treatment.

*In vivo* anti-metastasis efficacy was then investigated using H&E staining of lungs and livers. In the staining images of lungs, metastatic tumor nodules were detected in AMP, AMP + US, PBS, PBS + US and AMPS groups, but not in AMPS + US groups (Figure 5B). In the liver tissues, metastatic tumor nodules were observed in PBS, PBS + US, AMP, AMP + US and AMPS groups, while which were not observed in AMPS + US group (Figure 5C). These results suggested that the tumor metastasis in lungs and livers could be obviously inhibited in AMPS + US group. The anti-metastasis efficacy in AMPS + US group was higher than that in AMPS group, which shoud be due to the lower levels of remaining tumor cells after SDT and CDT effect. The combinational action of CDT and SDT killed the tumor cells and the released SIS3 inhibited the migration of tumor cells, which contributed to the inhibition of tumor metastasis.

## 4 Conclusion

In summary, we have developed a ALG-based hybrid hydrogel containing a metastasis inhibitor for combinational SDT-CDT treatment and tumor metastasis inhibition. The hydrogels (AMPS) were formed via chelation of ALG by Ca^2+^ in tumor microenvironment, which were composed of MnO_2_ nanoparticles as the CDT agents, an organic polymer as the sonosensitizer, and a metastasis inhibitor (SIS3). Under US irradiation of tumor sites, AMPS mediated both CDT and SDT via Fenton-like reaction and sonodynamic effect to effectively kill tumor cells. Moreover, AMPS hydrogels released SIS3 into tumor microenvironment to inhibit the metastasis of tumor cells. The therapeutic efficacy of AMPS hydrogels was satisfactory because the growths of subcutaneous 4T1 tumors were effectively inhibited and the tumor metastasis in lungs and livers were also entirely abolished. Owing to the local drug release property of hydrogels, this therapeutic platform showed good biosafety. In view of good tissue penetration depths of SDT and CDT, such a therapeutic method may be explored for treatment of other tumor types, and even deep-seated tumors.

## Data Availability

The original contributions presented in the study are included in the article/Supplementary Material, further inquiries can be directed to the corresponding authors.
