# Supplementary material for: Drug-loaded hybrid hydrogels for sonodynamic-chemodyanmic therapy and tumor metastasis suppression
Source: Front Bioeng Biotechnol. 2023 Sep 18;11:1281157. doi: 10.3389/fbioe.2023.1281157 (PMC10544978; doi:10.3389/fbioe.2023.1281157)
Supplement: Supplementary file 1 [file Table1.DOCX]

Supplementary Material

Drug-loaded hybrid hydrogels for sonodynamic-chemodyanmic therapy and tumor metastasis suppression

Xiaoying Wang^1#^, Liyun Zhu^2#^, Jianhui Zhou^2^, Lingzhou Zhao^3*^, Jingchao Li^2*^, Changcun Liu^3*^

^1^Office of Hospital Infection and Disease Control and Prevention, Shanghai General Hospital, Shanghai Jiao Tong University School of Medicine, Shanghai 200080, China

^2^College of Biological Science and Medical Engineering, Donghua University, Shanghai 201620, China

^3^Department of Nuclear Medicine, Shanghai General Hospital, Shanghai Jiao Tong University School of Medicine, Shanghai 200080, China

^#^**These authors contributed equally to this work.**

***Correspondence:**zlz-330@163.com (L. Zhao), jcli@dhu.edu.cn (J. Li), changcunwlj@163.com (C. Liu)

## Supplementary Figures


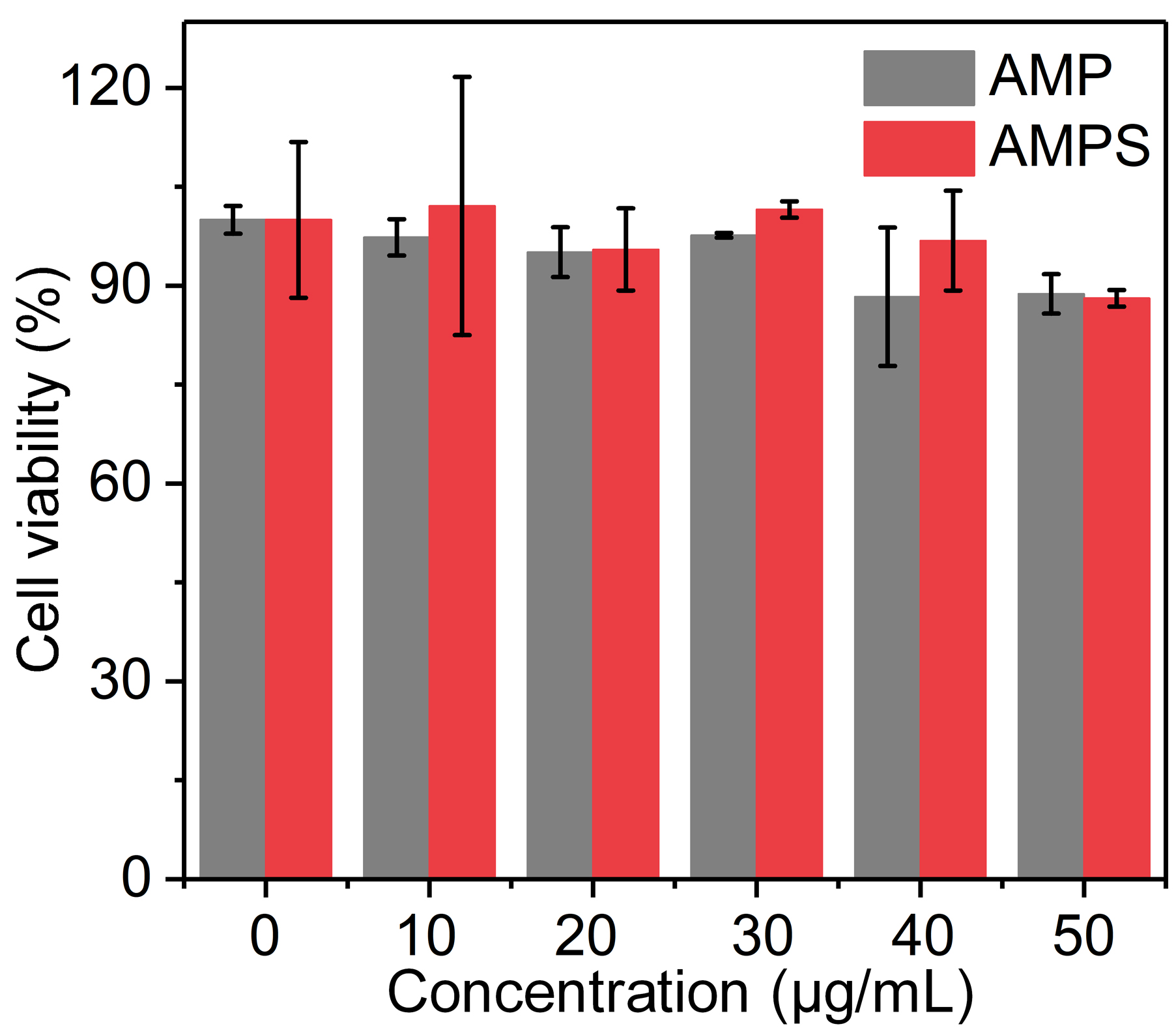


**Supplementary Figure 1.** Cell viability of 4T1 cells after incubation with AMP and AMPS hydrogels for 24 h.


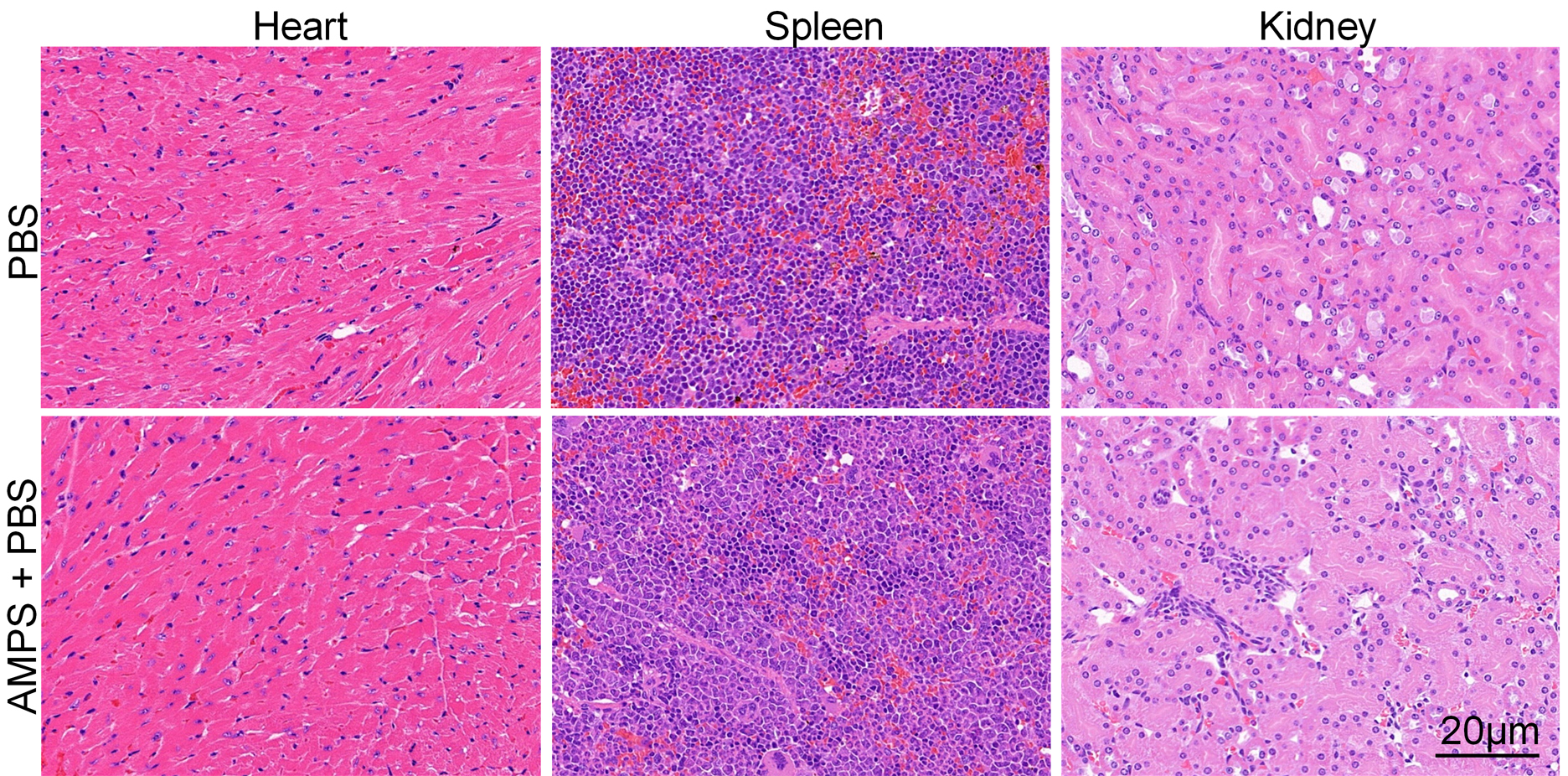


**Supplementary Figure 2.** H&E staining images of heart, spleen and kidney in PBS and AMPS + US groups.
